# Developing a novel evaluation model to assess the performance of base hospitals in China National Stroke Screening Surveys

**DOI:** 10.1371/journal.pone.0336499

**Published:** 2025-11-26

**Authors:** Xuedong Liu, Jian Cao, Ou Jiang

**Affiliations:** 1 Department of Medical Administration, The First People’s Hospital of Neijiang, Shizhong, Neijiang, People's Republic of China; 2 School of Public Health, Chongqing Medical University, Shapingba, Chongqing, People's Republic of China; West China Hospital, Sichuan University, CHINA

## Abstract

**Background:**

The China National Stroke Screening Surveys (CNSSS), a decade-long national public health initiative, aims to reduce stroke morbidity and mortality through early detection and intervention. This study develops a novel evaluation model to systematically assess the performance of base hospitals (BHS) participating in the CNSSS program in Sichuan Province, China.

**Methods:**

Sixteen BHS in Sichuan Province participated in the CNSSS program in 2024. We utilized eleven performance indicators to develop the evaluation model. Subjective weights were derived from scores assigned to indicators, while objective weights were calculated using the Entropy Weight Method (EWM). A Multiplicative Synthesis with Normalization (MSN) method was adopted to generate combined weights. Based on the subjective, objective, and combined weights, we generated weighted data matrices, determined the corresponding BHS rankings separately, and compared BHS performance in CNSSS implementation across these three ranking systems.

**Results:**

Among the evaluation indicators, Task Completion Rate received the highest subjective weight (0.3125), whereas Intervention Rate dominated both objective (0.1594) and combined (0.2303) weights. Notable weight changes were observed: Task Completion Rate exhibited the largest reduction (−92.45%) from subjective to objective weights, followed by Age Deviation Degree (−76.11%), Follow-up Completion Rate (−73.31%), and Hypertension Awareness Rate (−3.31%). Conversely, Diabetes Detection Rate displayed the most significant increase (+313.65%), followed by Dyslipidemia Detection Rate (+249.44%), Hypertension Detection Rate (+227.79%), Stroke High-Risk Detection Rate (+119.52%), Stroke High-Risk Intervention Rate (+107.90%), Intervention Rate (+48.77%), and Risk Factor Control Rate (+42.52%). BHS A and B ranked top 3 across all weighting methodologies. BHS A ranked first under original, subjective and combined weights, while BHS D led in the objective ranking. Compared to the original methodology, the combined weighting methodology has the highest discrimination degree (0.1166).

**Conclusions:**

Weighting methodologies significantly influence BHS performance evaluations. Subjective approaches emphasize expert expertise, whereas objective methods prioritize data variability. Compared to single weighting method, combined weighting effectively balances discrepancies between expert subjective priorities and data-driven objectivity, thereby addressing limitations of single-method designs. For the CNSSS program, our model underscores the need to shift quality focus from high-risk screening to targeted management, including timely post-screening interventions and effective risk factor control. These targeted interventions are expected to significantly reduce stroke incidence, recurrence, and mortality among screened populations, while enhancing the overall quality of the CNSSS program in the region.

## Introduction

According to the 2021 Global Burden of Disease (GBD) study, stroke remains the second leading cause of death and the third leading cause of death and disability combined among non-communicable diseases (NCD_S_) globally [1]. Between 1990 and 2021, there was a marked increase in the number of people affected by stroke worldwide. Specifically, stroke incidence rose by 70% (95% Uncertainty Interval [UI]: 66–75), mortality by 44% (95% UI: 32–56), and disability-adjusted life years (DALY_S_) by 32% (95% UI: 22–43) [2]. The majority of the global stroke burden is concentrated in low-income and lower-middle-income countries (LMIC_S_). It is estimated that the global cost of stroke exceeds 890 billion USD annually. Furthermore, stroke mortality is projected to increase by 50% from 2020 to 2050, with DALY_S_ rising from 144.8 million (95% UI: 133.9–156.9 million) in 2020 to 189.3 million (95% UI: 161.8–224.9 million) in 2050 [3].

In China, the most recent “Brief Report on Stroke Prevention and Treatment in China, 2021” revealed that stroke ranked as the leading cause of death and disability among the Chinese population [4]. The epidemiology of stroke in China exhibits several distinct characteristics: 1) Ischemic stroke incidence has shown a gradual upward trend, increasing from 0.129% in 2010 to 0.145% in 2019, while hemorrhagic stroke incidence has shown a gradual downward trend, decreasing from 0.061% in 2010 to 0.045% in 2019; 2) The prevalence of ischemic stroke has risen from 1.100% in 2010 to 1.256% in 2019, whereas the prevalence of hemorrhagic stroke has declined from 0.232% in 2010 to 0.215% in 2019; 3) The crude mortality rate of stroke among rural residents has shown an upward trend with fluctuations between 2010 and 2019 and has consistently been higher than that of urban residents during the same period; 4) The DALY_S_ for ischemic stroke have declined from 1.209% in 2010 to 1.148% in 2019, while those for hemorrhagic stroke have shown a notable downward trend, decreasing from 1.167% in 2010 to 1.142% in 2019; 5) The average inpatient medical expenditures per ischemic and hemorrhagic stroke patient increased by 37% and 82%, respectively, from 2010 to 2019 [4]. These findings underscore that stroke has become a major public health challenge in China.

As the largest developing country globally, China accounts for one-fifth of the world’s population. The burden of stroke in China is intensifying due to population aging and inadequate control of stroke-related risk factors such as hypertension, diabetes, and dyslipidemia [5]. In response to these urgent challenges, the Chinese government has prioritized stroke prevention and treatment within the Healthy China Initiative and the Healthy China 2030 campaign [6]. In 2011, the China Stroke Prevention Project Committee (CSPPC) was established by the then Ministry of Health (CMH), with the goal of reducing stroke incidence by less than 5% and decreasing mortality from cardiovascular and cerebrovascular diseases by 10% by 2020 [6]. To achieve these objectives, the CSPPC has implemented several measures, including formulating stroke prevention and treatment guidelines, accrediting a number of demonstration stroke centers, higher-level stroke centers, stroke prevention and treatment centers, and stroke map hospitals, as well as organizing national stroke education and training for healthcare professionals [6]. To further enhance the concept of “prevention and treatment coordination”, the CSPPC launched the China National Stroke Screening Survey (CNSSS) in 2011 [4,6]. The CNSSS is an ongoing community-based screening program funded by the central government, targeting residents aged 40 and above across all provinces in mainland China [7].

Hospitals have been a key force in implementing the CNSSS program. By 2021, the CSPPC had designated 327 tertiary hospitals as stoke screening and prevention base hospitals (BHS) [4]. The responsibilities of BHS include: 1) Pre-hospital promotion of an integrated prevention and treatment model, focusing on screening high-risk stroke populations and providing coordinated emergency care for acute stroke patients; 2) In-hospital implementation of high-risk stroke screening and multidisciplinary joint diagnosis and treatment; and 3) Post-hospital follow-up and intervention for patients [4]. Moreover, BHS are tasked with training and guiding healthcare professionals from community health service centers and township health centers to conduct high-risk stroke population screening, follow-up, and risk factor intervention. Under the supervision and guidance of BHS, the CNSSS completed 1.256 million stroke screening and prevention activities, identified 268,000 high-risk stroke individuals, and implemented 662,000 follow-up and intervention in 2020.

The quality of BHS work directly impacts the effectiveness of the CNSSS program. High-quality BHS work ensures accurate identification, timely follow-up, and appropriate intervention for high-risk stroke populations. To evaluate BHS performance, the Office of the CSPPC constructed an evaluation matrix consisting of 11 indicators [8], aiming to improve the quality of stroke screening and prevention among residents aged 40 and older. However, the existing methodology of calculating the total score for each BHS-by simply aggregating the scores of individual indicators (assigning equal weights to each)-may, to some extent, introduce biases and lead to distorted evaluation results. It is widely recognized that incorporating weights is crucial for obtaining scientifically robust and accurate evaluation results [9]. Our literature review revealed that no evaluation model has been developed in China to assess the performance of BHS in conducting the CNSSS program.

To address this gap, we developed a novel combined-weighted evaluation model, integrating subjective weights from expert judgment and the objective weights from the Entropy Method, to systematically evaluate performance disparities among BHS in conducting the CNSSS program. Firstly, subjective weights of the indicators were determined by the scores given by experts from the CSPPC; secondly, the Entropy Method was utilized to ascertain the objective weights of the indicators; thirdly, the multiplicative synthesis with normalization (MSN) method was applied to calculate the combined weights of the indicators; lastly, the performance of each BHS participating in the CNSSS program was re-ranked based on the combined weighted scores (CWS). The findings of this study may: 1) Fill the literature gap regarding the theoretical implications of weight determination for evaluating BHS performance during CNSSS implementation; 2) Offer practical insights to enhance the quality of the CNSSS program; 3) Provide useful experience in conducting performance evaluation of similar programs.

## Methods and materials

### Study design

This study was a retrospective cross-sectional study conducted at the First People’s Hospital of Neijiang, Sichuan Province, China. The study did not involve human participants, nor did it use human tissue samples or human biomedical information (e.g., name, ID number, admission number, diagnostic results, examination data). Therefore, ethical approval were waived by the Ethics Committee of the First People’s Hospital of Neijiang.

### Data sources

**Fig 1:**
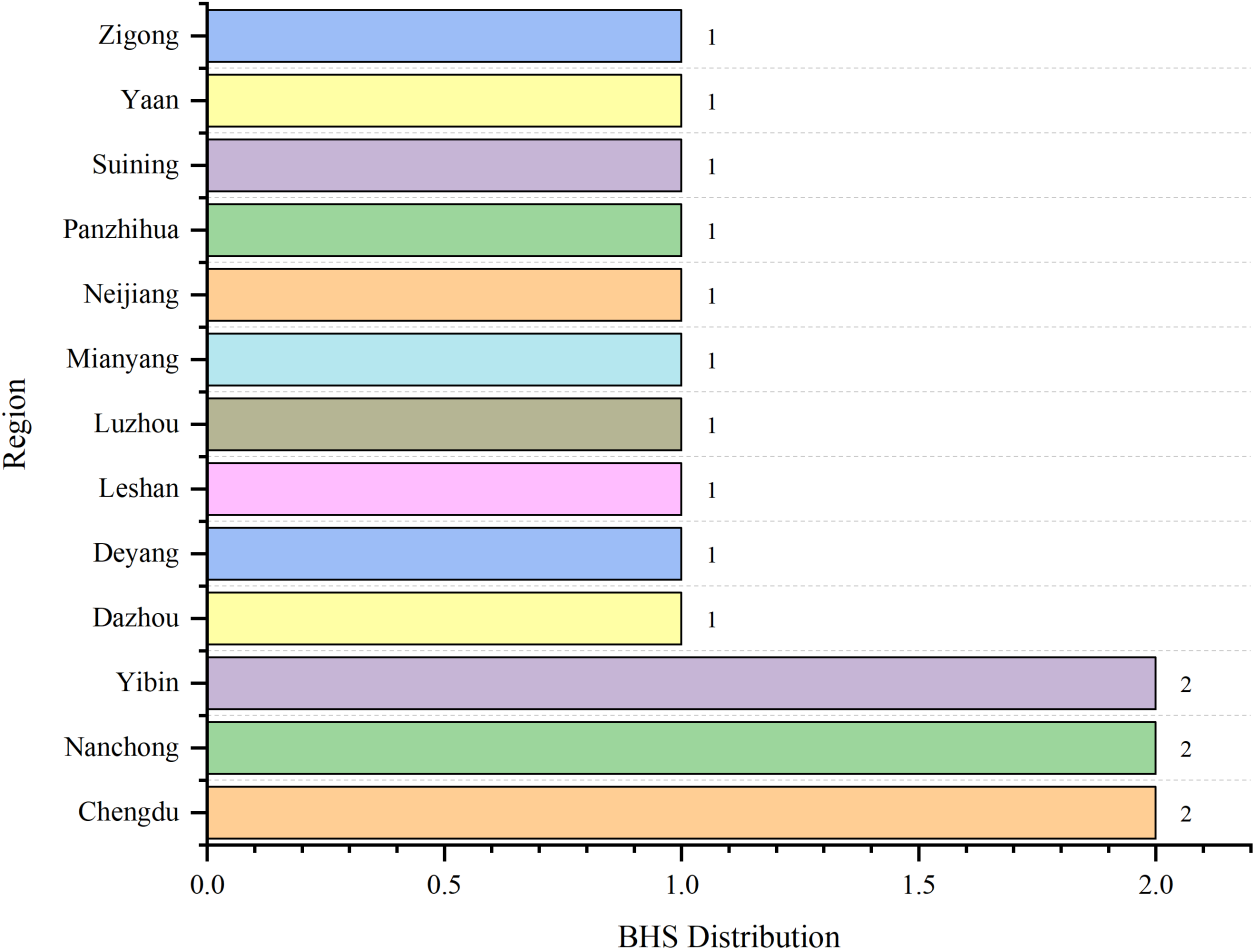
The geographical distribution of BHS in Sichuan Province. BHS: base hospitals.

In 2024, sixteen BHS across Sichuan Province, participated in the CNSSS program. In March, 2025, the Office of the CSPPC issued the 2024 annual quality control reports on BHS participating in the CNSSS program. The data for this study were derived from these reports, and all data were complete, valid, and endorsed by the Office of the CSPPC. All BHS were tertiary A general hospitals (the highest level in China’s hospital ranking system), distributed across 13 municipalities in Sichuan Province. Specifically, Chengdu (hospitals E and I), Nanchong (hospitals A and B), and Yibin (hospitals L and O) each occupied two BHS, while Dazhou (hospital N), Deyang (hospital J), Leshan (hospital F), Luzhou (hospital G), Mianyang (hospital D), Neijiang (hospital K), Panzhihua (hospital P), Suining (hospital H), Yaan (hospital M), and Zigong (hospital C) each had one BHS. The geographical distribution of these BHS is shown in Fig 1.

### Indicators

The Office of the CSPPC proposed eleven evaluation indicators to assess the annual performance of BHS. Specifically, Task Completion Rate, Risk Factor Control Rate, Hypertension Awareness Rate, Stroke High-Risk Intervention Rate, Intervention Rate, and Follow-up Completion Rate were categorized as positive indicators, whereas Stroke High-Risk Detection Rate, Hypertension Detection Rate, Diabetes Detection Rate, Dyslipidemia Detection Rate, and Age Deviation Degree were defined as moderate indicators. The detailed evaluation matrix for BHS performance assessment is presented in Table 1.

**Table 1:** The evaluation matrix for BHS performance assessment.

Order	Indicator	Assigned score	Calculation Method	Scoring Criteria	Attributes
1	Task Completion Rate (X1)	35 points	(Reviewed records/ Assigned workload) × 100%	100% ≤ P (35 points) 95% ≤ P < 100% (30 points) 90% ≤ P < 95% (25 points) 85% ≤ P < 90% (20 points) 0% ≤ P < 85% (15 points) 0% = P (0 points)	Positive
2	Risk Factor Control Rate (X2)	12 points	(Controlled cases/ Total cases) × 100%	(National Standards) = P (12 points) (National Standards)×10% ≤ P <(National Standards) (9 points) (National Standards)×20% ≤ P <(National Standards)×10% (6 points) (National Standards)×30% ≤ P <(National Standards)×20% (3 points) Others (0 points)	Positive
3	Stroke High-Risk Detection Rate (X3)	6 points	(High-risk individuals/ Screened individuals) × 100%	26% < P (3 points) 20% ≤ P ≤ 26% (6 points) 15% ≤ P < 20% (4 points) 10% ≤ P < 15% (3 points) P < 10% (0 points)	Moderate
4	Hypertension Awareness Rate (X4)	6 points	(Known hypertension cases/ Total hypertension cases) × 100%	(National Standards) = P (6 points) (National Standards)×10% ≤ P <(National Standards) (4 points) (National Standards)×20% ≤ P <(National Standards)×10% (3 points) (National Standards)×30% ≤ P <(National Standards)×20% (2 points) (National Standards)×40% ≤ P <(National Standards)×30% (1 point) Others (0 points)	Positive
5	Hypertension Detection Rate (X5)	3 points	(Hypertension cases/ Screened individuals) × 100%	(Sichuan Provincial Standards) = P (3 points) (Sichuan Provincial Standards)×10% ≤ P <(Sichuan Provincial Standards) (2 points) (Sichuan Provincial Standards)×20% ≤ P <(Sichuan Provincial Standards)×10% (1 point) Others (0 points)	Moderate
6	Diabetes Detection Rate (X6)	3 points	(Diabetes cases/ Screened individuals) × 100%	(Sichuan Provincial Standards) = P (3 points) (Sichuan Provincial Standards)×10% ≤ P <(Sichuan Provincial Standards) (2 points) (Sichuan Provincial Standards)×20% ≤ P <(Sichuan Provincial Standards)×10% (1 point) Others (0 points)	Moderate
7	Dyslipidemia Detection Rate (X7)	3 points	(Dyslipidemia cases/ Screened individuals) × 100%	(Sichuan Provincial Standards) = P (3 points) (Sichuan Provincial Standards)×10% ≤ P <(Sichuan Provincial Standards) (2 points) (Sichuan Provincial Standards)×20% ≤ P <(Sichuan Provincial Standards)×10% (1 point) Others (0 points)	Moderate
8	Stroke High-Risk Intervention Rate (X8)	8 points	(Intervened high-risk individuals/ Total high-risk individuals) × 100%	(National Standards) = P (8 points) (National Standards)×10% ≤ P <(National Standards) (6 points) (National Standards)×20% ≤ P <(National Standards)×10% (3 points) (National Standards)×30% ≤ P <(National Standards)×20% (1 point) Others (0 points)	Positive
9	Intervention Rate (X9)	12 points	(Intervened individuals/ Assigned workload) × 100%	85% ≤ P (12 points) 70% ≤ P < 85% (10 points) 50% ≤ P < 70% (7 points) 30% ≤ P < 50% (5 points) P < 30% (0 points)	Positive
10	Follow-up Completion Rate (X10)	12 points	(2023 follow-ups completed/ 2023 follow-ups required) × 100%	100% ≤ P (12 points) 85% ≤ P < 100% (10 points) 70% ≤ P < 85% (7 points) 50% ≤ P < 70% (5 points) P < 50% (0 points)	Positive
11	Age Deviation Degree (X11)	12 points	[(The proportion of the population in each age group to the total number of screened individuals-Proportion of each age group from the 7th National Census)/(Proportion of each age group from the 7th National Census)] × 100%	(National Standards) = P (12 points) (National Standards)×10% ≤ P <(National Standards) (9 points) (National Standards)×20% ≤ P <(National Standards)×10% (6 points) (National Standards)×30% ≤ P <(National Standards)×20% (3 points) Others (0 points)	Moderate

### Procedures for the construction of the evaluation model

#### Data processing.

Data were processed using Excel 2024. All calculations were completed using functions built in Excel.

#### Determining the weights of the evaluation indicators.

Weights are widely recognized as a crucial element in multi-criteria decision-making process due to its significant influence on the outcomes of comprehensive evaluations [10,11]. The selection of appropriate weighting methodologies directly affects the accuracy and reliability of evaluation results [12]. Currently, weighting methodologies are categorized into three types: subjective, objective, and combined (integrated) [10]. To ensure the robustness and trustworthiness of evaluation results, it is recommended to employ multiple methodologies when determining weights [13]. Given these considerations, our study adopted a combined weighting approach to determine the weights of the evaluation indicators.

#### Subjective weights.

CSPPC experts evaluated the relative importance of each evaluation indicator and assigned corresponding scores. The subjective weights (W_S_) for the evaluation indicators were derived from these scores using Equation (1).


$$W_{j}^{s}=\frac{S_{j}}{\sum_{j=1}^{m}S_{j}}$$ (1)

where m denotes the number of indicators and $S_{j}$ corresponds to the score assigned to the j-th indicator.

#### Objective weights.

Various methodologies can be applied to calculate the objective weights for evaluation indicators, such as principal component analysis (PCA) [14], Entropy Weight Method (EWM) [15], and coefficient of variation (CV) [16]. In this study, we adopted the EWM to determine the objective weights for the evaluation indicators. Detailed procedures were as follows:

1. The original data matrix $X_{ij}$ was standardized using Equations (2)–(4) [17], where i = 1,2,…,n; j = 1,2,…,m; n represents the number of BHS; m represents the number of evaluation indicators.

Positive indicators:


$$Y_{ij}=\frac{X_{ij}-\min(X_{ij})}{\max(X_{ij})-\min(X_{ij})}$$ (2)

Negative indicators:


$$Y_{ij}=\frac{\max(X_{ij})-X_{ij}}{\max(X_{ij})-\min(X_{ij})}$$ (3)

Moderate indicators:


$$Y_{ij}=1-\frac{\left|X_{ij}-{\overset{―}{X}}_{j}\right|}{\max\left|X_{ij}-{\overset{―}{X}}_{j}\right|}$$ (4)

2. The standardized data matrix was added 0.001 to eliminate the influence of zero values.

$$Y_{ij}^{\prime }=Y_{ij}+0.001$$ (5)

3. The value of $P_{ij}$ was calculated.

$$P_{ij}=\frac{Y_{j}^{\prime }}{\sum_{j=1}^{n}Y_{j}^{\prime }}$$ (6)

4. The value of $E_{j}$ was calculated.

$$E_{j}=-\frac{\text{1}}{Ln(n)}\sum_{i=1}^{m}P_{ij}\times Ln({P_{i}}_{j})$$ (7)

5. The objective weights were calculated.

$$W_{j}^{o}=\frac{1-E_{j}}{m-\sum_{j=1}^{m}E_{j}}$$ (8)

#### Combined weights.

After obtaining the subjective and objective weights of the evaluation indicators via the aforementioned approaches, we then integrated these weights appropriately to obtain the combined weights for the evaluation indicators. Commonly applied weight combination methodologies include least squares linear fusion (LSLF), MSN, and multi-weight fusion based on maximum membership degree (MWFMMD) [18]. In our study, we assumed equal importance of subjective and objective weights [18], aimed to enlarge the disparities in evaluation indicators to enhance differentiation between evaluation results, and ensured interpretation and horizontal comparability of results through normalization [19]. Thus, we utilized the MSN methodology to calculate the combined weights for the evaluation indicators. The main calculation function was as follows:


$$W_{j}^{c}=\frac{\prod_{l=1}^{L}W_{j}^{l}}{\sum_{j=1}^{m}\prod_{l=1}^{L}W_{j}^{l}}$$ (9)

where $W_{j}^{c}$ denotes the combined weight of the j-th indicator; ι=1, 2,...L, L represents the number of weighting methodologies; $\overset{L}{\underset{l=1}{∐}}W_{j}^{l}$ represents the product of weights of the j-th indicator using L weighting methodologies; and $\sum_{j=1}^{m}\overset{L}{\underset{l=1}{∐}}W_{j}^{l}$ represents the sum of such products across all indicators.

#### Calculating the total weighted scores (TWS) and rankings for each BHS.

The original data matrix was recalculated according to the subjective, objective, and combined weights of the evaluation indicators, respectively. Subsequently, the performance of each BHS conducting the CNSSS program was re-ranked based on the TWS values. The calculation functions were as follows:


$$X_{ij}^{s}=W_{j}^{s}\times X_{ij},\;X_{ij}^{o}=W_{j}^{o}\times X_{ij},\;X_{ij}^{w}=W_{j}^{c}\times X_{ij}$$ (10)

$$TWS_{s}=\sum_{j=1}^{m}X_{j}^{w},\;TWS_{o}=\sum_{j=1}^{m}X_{j}^{w},\;TWS_{c}=\sum_{j=1}^{m}X_{j}^{w}$$ (11)

where j = 1,2,…,m, m represents the number of evaluation indicators; $X_{j}^{w}$ represents the weighted score of the j-th indicator; and TWS_S_, TWS_O_, and TWS_C_ represent the total weighted scores for each BHS, respectively.

#### Comparison of different evaluation methodologies.

Setting the original scores as the baseline, three weighting methodologies were compared using two indicators (head-tail consistency rate and discrimination degree) proposed by professor Yu LP, et al [11,20]. The head-tail consistency rate measures the proportion of top 20% and bottom 20% BHS consistently identified by different evaluation methodologies. Its value ranges between 0 and 1, with a higher value indicating stronger consistency across methodologies. The discrimination degree quantifies the ability of an evaluation methodology to magnify performance differences between BHS. A higher value means the methodology can more clearly distinguish “superior” from “inferior” BHS. The calculation functions were as follows:

Head-tail consistency rate:


$$S=\frac{x+y}{0.4\times n}$$ (12)

where x represents the number of BHS consistently ranked in the top 20%; y denotes the number of BHS consistently ranked in the bottom 20%; n represents the total number of BHS.

Discrimination degree:


$$D=\frac{\sum_{i=1}^{n-1}\sqrt{{\left(V_{i+1}-V_{i}\right)}^{2}+1}}{\sqrt{2n^{2}-2n-1}},\;V_{i}=n\times \left(1-\frac{\left|V_{i}^{\prime }-V_{1}^{\prime }\right|}{V_{1}^{\prime }-V_{n}^{\prime }}\right)$$ (13)

where n represents the number of BHS; 1 ≤ i ≤ n; $V_{i}^{\prime }$ represents the original score, $V_{1}^{\prime }$ is the largest score and $V_{n}^{\prime }$ is the smallest score; $V_{i}$ represents the standardized value corresponding to $V_{i}^{\prime }$.

## Results

### Subjective weights of evaluation indicators

The highest subjective weight was assigned to Task Completion Rate, with a value of 0.3125. Four evaluation indicators, including Risk Factor Control Rate, Intervention Rate, Follow-up Completion Rate, and Age Deviation Degree, received the second-highest and equal subjective weights (0.1071), reflecting that experts perceived them as equally important. Followed by Stroke High-Risk Intervention Rate (0.0714), Hypertension Awareness Rate, and Stroke High-Risk Detection Rate (0.0536). Three evaluation indicators: Hypertension Detection Rate, Diabetes Detection Rate, and Dyslipidemia Detection Rate were allocated the smallest and equal weights (0.0268). Details are shown in Table 2.

**Table 2:** The subjective weights of the evaluation indicators.

Order	Indicator	Subjective weight
1	Task Completion Rate (X1)	0.3125
2	Risk Factor Control Rate (X2)	0.1071
3	Intervention Rate (X9)	0.1071
4	Follow-up Completion Rate (X10)	0.1071
5	Age Deviation Degree (X11)	0.1071
6	Stroke High-Risk Intervention Rate (X8)	0.0714
7	Stroke High-Risk Detection Rate (X3)	0.0536
8	Hypertension Awareness Rate (X4)	0.0536
9	Hypertension Detection Rate (X5)	0.0268
10	Diabetes Detection Rate (X6)	0.0268
11	Dyslipidemia Detection Rate (X7)	0.0268

### Objective weights of evaluation indicators

Contrasts to the subjective weights, the Intervention Rate received the highest objective weight, with a value of 0.1594. Followed by Risk Factor Control Rate (0.1527), Stroke High-Risk Intervention Rate (0.1485), Stroke High-Risk Detection Rate (0.1176), Diabetes Detection Rate (0.1108), Dyslipidemia Detection Rate (0.0936), Hypertension Detection Rate (0.0878), Hypertension Awareness Rate (0.0518), Follow-up Completion Rate (0.0286), and Age Deviation Degree (0.0256). Notably, the Task Completion Rate obtained the smallest weight, with a value of 0.0236. Details are displayed in Table 3.

**Table 3:** The objective weights of the evaluation indicators.

Order	Indicator	Objective weight
1	Task Completion Rate (X1)	0.0236
2	Risk Factor Control Rate (X2)	0.1527
3	Intervention Rate (X9)	0.1594
4	Follow-up Completion Rate (X10)	0.0286
5	Age Deviation Degree (X11)	0.0256
6	Stroke High-Risk Intervention Rate (X8)	0.1485
7	Stroke High-Risk Detection Rate (X3)	0.1176
8	Hypertension Awareness Rate (X4)	0.0518
9	Hypertension Detection Rate (X5)	0.0878
10	Diabetes Detection Rate (X6)	0.1108
11	Dyslipidemia Detection Rate (X7)	0.0936

### Combined weights of evaluation indicators

Among the eleven evaluation indicators, the Intervention Rate obtained the highest combined weight (0.2303), followed by the Risk Factor Control Rate (0.2207), Stroke High-risk Intervention Rate (0.1430), and Task Completion Rate (0.0995). Six indicators: Follow-up Completion Rate, Diabetes Detection Rate, Hypertension Awareness Rate, Age Deviation Degree, Dyslipidemia Detection Rate, and Hypertension Detection Rate exhibited relatively small and stable combined weights, with values of 0.0413, 0.0401, 0.0375, 0.0370, 0.0339, and 0.0318, respectively. Details are presented in Table 4.

**Table 4:** The combined weights of the evaluation indicators.

Order	Indicator	Objective weight
1	Task Completion Rate (X1)	0.0995
2	Risk Factor Control Rate (X2)	0.2207
3	Intervention Rate (X9)	0.2303
4	Follow-up Completion Rate (X10)	0.0413
5	Age Deviation Degree (X11)	0.0370
6	Stroke High-Risk Intervention Rate (X8)	0.1430
7	Stroke High-Risk Detection Rate (X3)	0.0850
8	Hypertension Awareness Rate (X4)	0.0375
9	Hypertension Detection Rate (X5)	0.0318
10	Diabetes Detection Rate (X6)	0.0401
11	Dyslipidemia Detection Rate (X7)	0.0339

### Comparison of subjective, objective, and combined weights of evaluation indicators

**Fig 2:**
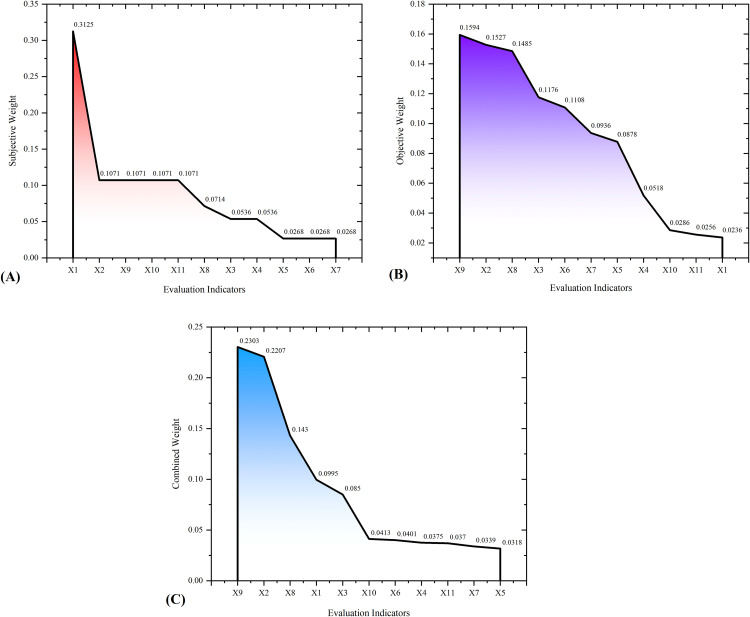
Comparison of subjective, objective, and combined weights of evaluation indicators. (A) The results of subjective weights. (B) The results of objective weights. (C) The results of combined weights. X1: Task Completion Rate; X2: Risk Factor Control Rate; X3: Stroke High-Risk Detection Rate; X4: Hypertension Awareness Rate; X5: Hypertension Detection Rate; X6: Diabetes Detection Rate; X7: Dyslipidemia Detection Rate; X8: Stroke High-Risk Intervention Rate; X9: Intervention Rate; X10: Follow-up Completion Rate; X11: Age Deviation Degree.

With subjective weights as the baseline, four indicators: Task Completion Rate, Follow-up Completion Rate, Age Deviation Degree, and Hypertension Awareness Rate exhibited declining objective weights. Among these, Task Completion Rate displayed the most significant reduction (92.45%; 0.3125 vs. 0.0236), whereas Hypertension Awareness Rate remained relatively stable, with only a 3.36% decrease (0.0536 vs. 0.0518). Conversely, seven indicators: Risk Factor Control Rate, Intervention Rate, Stroke High-Risk Intervention Rate, Stroke High-Risk Detection Rate, Hypertension Detection Rate, Diabetes Detection Rate, and Dyslipidemia Detection Rate demonstrated increasing trends. Notably, Diabetes Detection Rate, Dyslipidemia Detection Rate, Hypertension Detection Rate, and Stroke High-Risk Detection Rate experienced the largest proportional increases: 313.43% (0.0268 vs. 0.1108), 249.25% (0.0268 vs. 0.0936), 227.61% (0.0268 vs. 0.0878), and 119.40% (0.0536 vs. 0.1176), respectively. Detailed results are presented in Fig 2.

### Scores and rankings of BHS under different evaluation methodologies

The rankings of BHS were determined based on the respective scores corresponding to each BHS. The top three BHS under the original methodology were BHS A,B, and C, with original scores (99.0000 vs. 96.2500 vs. 91.0000). However, the order under the subjective, objective, and combined methodologies were different, with subjective: A, B, G (16.3824 vs. 15.9006 vs. 15.4345); objective: D, B, A (7.6998 vs. 7.6021 vs. 7.3770); combined: A, B, D (11.4458 vs. 11.2582 vs. 11.0085). Similarly, the bottom three BHS varied by methodology, with original: N, O, P (62.8000 vs. 56.8000 vs. 56.0000); subjective: O, P, J (12.6569 vs. 12.4106 vs. 12.3149); objective: P, M, O (2.1406 vs. 2.1389 vs. 2.0551); and combined: M, O, P (4.6854 vs. 4.5789 vs. 4.5001). Detailed results are presented in Table 5.

**Table 5:** Scores and rankings of BHS under different evaluation methodologies.

Original Methodology			Subjective Methodology			Objective Methodology			Combined Methodology		
Hospital Code	Original Score	Ranking	Hospital Code	Subjective Score	Ranking	Hospital Code	Objective Score	Ranking	Hospital Code	Combined Score	Ranking
A	99.0000	1	A	16.3824	1	D	7.6998	1	A	11.4458	1
B	96.2500	2	B	15.9006	2	B	7.6021	2	B	11.2582	2
C	91.0000	3	G	15.4345	3	A	7.3770	3	D	11.0085	3
D	90.0000	4	F	15.3810	4	C	6.7346	4	I	10.3172	4
E	87.0000	5	C	15.2580	5	H	6.5609	5	E	9.9964	5
F	86.5000	6	E	15.2578	6	F	6.4842	6	G	9.9930	6
G	85.7500	7	I	15.1131	7	E	6.3922	7	H	9.9650	7
H	84.0000	8	H	15.0704	8	G	6.3314	8	F	9.8955	8
I	79.7500	9	D	14.6593	9	I	6.2837	9	C	9.8600	9
J	76.0000	10	L	13.2996	10	J	6.2666	10	J	9.1810	10
K	67.4000	11	K	13.2569	11	K	2.7206	11	K	4.9734	11
L	66.8000	12	M	13.2085	12	L	2.5531	12	L	4.8145	12
M	63.2000	13	N	12.9249	13	N	2.4553	13	N	4.7988	13
N	62.8000	14	O	12.6569	14	P	2.1406	14	M	4.6854	14
O	56.8000	15	P	12.4106	15	M	2.1389	15	O	4.5789	15
P	56.0000	16	J	12.3149	16	O	2.0551	16	P	4.5001	16

### Comparison of different evaluation methodologies

**Fig 3:**
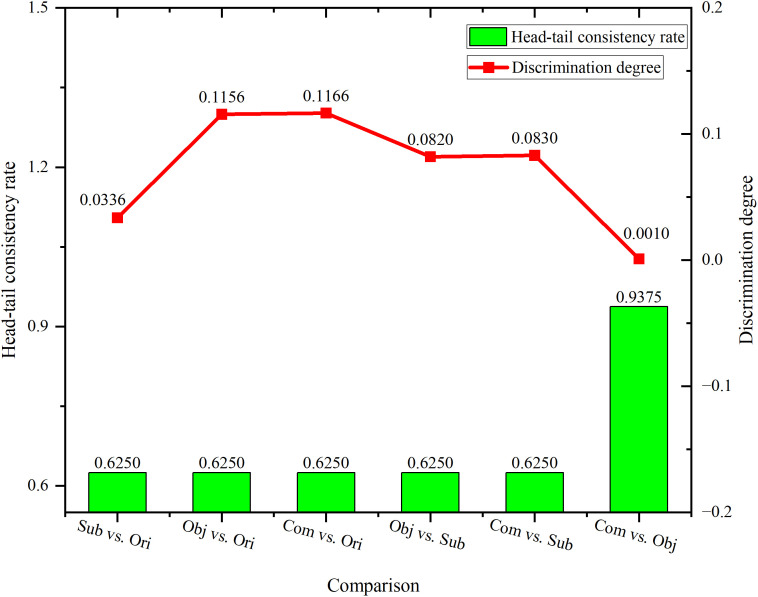
Comparison of original, subjective, objective, and combined evaluation methodologies. Ori: Original; Sub: Subjective; Obj: Objective; Com: Combined.

For the first five comparison pairs (Subjective vs. Original, Objective vs. Original, Combined vs. Original, Objective vs. Subjective, and Combined vs. Subjective), the head-tail consistency rate remained stable at 0.6250. This indicates consistent alignment in the head-tail distribution patterns of these evaluation methodologies. Notably, in the Combined vs. Objective pair, this rate surged to the highest value of 0.9375, reflecting a substantially enhanced consistency between the combined and objective methodologies. The discrimination degree exhibited a more dynamic trend. It started at 0.0336 (Subjective vs. Original), then rose to the highest value 0.1166 (Combined vs. Original), suggesting a notable difference between the combined and original methodologies. Subsequently, it decreased to 0.0820 (Objective vs. Subjective), slightly rebounded to 0.0830 (Combined vs. Subjective), and finally dropped to 0.0010 (Combined vs. Objective). The extremely low discrimination degree in the Combined vs. Objective pair implied a high level of consistency between the two methodologies, which aligned with the highest head-tail consistency rate in this pair. Collectively, these results demonstrated that the combined methodology aligned well with the objective methodology in both head-tail consistency and discrimination degree, whereas the original methodology differed most from the combined approach. Detailed comparison results are presented in Fig 3.

## Discussion

### Necessity of establishing a model to evaluate the performance of BHS

In recent decades, the epidemiological characteristics of stroke have shifted dramatically, with incidence, mortality, and DALY_S_ increasing significantly [1]. These changes underscore the urgency of implementing effective stroke screening and prevention programs, such as the CNSSS, which relies heavily on BHS performance for high-quality implementation. Since 2011, the CNSSS has evolved into a national key public health initiative [8]. After over a decade of practice, developing a scientific and practical model to evaluate BHS performance in CNSSS implementation has become increasingly crucial. This model serves as an indispensable component of the CNSSS’s sustainable development [21]. Notably, however, to our knowledge, there remains a lack of literature that attempts to develop a model to assess BHS performance in CNSSS implementation. Previous studies have mainly focused on identifying stroke prevalence and stroke-related risk factors based on CNSSS data [22–24].

Our study is the first to develop a novel combined-weighted evaluation model, integrating experts’ subjective judgments and the objective Entropy Methodology, to systematically evaluate performance disparities among BHS in CNSSS implementation in Sichuan Province, China. The model addresses a critical limitation of the current CNSSS program: its practice of assigning equal weights to all evaluation indicators when assessing BHS performance. Our findings not only address the literature gap regarding the theoretical implications of weight determination for BHS performance evaluation in CNSSS implementation but also offer practical insights for enhancing the quality of the CNSSS program.

### Discrepancies between weights under different weighting methodologies

Subjective weighting methodologies, despite potential bias, reflect experts’ clinical and practical experience. This is exemplified by the highest subjective weight assigned to Task Completion Rate, an outcome that reflects administrative mandates and emphasizes compliance with the CNSSS program. In contrast, objective weighting methodologies mitigate bias by leveraging empirical data variability. This is evidenced by the Intervention Rate being assigned the highest objective weight, which surpasses its subjective counterpart, indicating that data-driven variability underscores its operational significance.Such complementarity aligns with debates in relevant literature: while subjective weighting methodologies (e.g., Analytic Hierarchy Process, AHP) excel at incorporating theoretical frameworks, objective methodologies (e.g., Entropy Method) specialize in capturing dynamic performance patterns [25]. Compared to single methodology, combined weighting methodologies balance discrepancies between experts’ subjective priorities and data-driven objectivity, thereby addressing limitations of single-method evaluations [26].

Our study identified notable discrepancies between subjective and objective weights of evaluation indicators. Compared to subjective weights, four indicators: Task Completion Rate, Follow-up Completion Rate, Age Deviation Degree, and Hypertension Awareness Rate exhibited reduced objective weights. The most pronounced finding is the 92.45% reduction in Task Completion Rate’s weight, dropping from 0.3125 (subjective) to 0.0236 (objective). This divergence highlights a fundamental difference in weight determination: subjective weights (derived from expert scoring) prioritize administrative compliance, whereas objective weights quantify indicators based on data discriminability. Specifically, indicators with higher entropy values are assigned higher objective weights [27], as they are independent of expert subjectivity. The low objective weight of Task Completion Rate reflects minimal performance variation across BHS, which had the smallest coefficient of variation (0.08), rendering it less informative for data-driven performance evaluations. This aligns with prior research showing that process-oriented indicators (e.g., Task Completion Rate) often exhibit ceiling effects in healthcare settings, limiting their ability to distinguish institutional work performance [28]. Importantly, the core mandate for BHS in the CNSSS program is to meet annual stroke screening and prevention targets and complete follow-up for these populations. BHS must use all accessible approaches to reach these mandatory missions, thus assigning higher weights to such indicators may fail to reflect genuine performance disparities among BHS.

In contrast, the objective weighting methodology (Entropy Method) prioritizes indicators with higher variation degrees (lower information entropy), as they better distinguish discrepancies between evaluated objects [29]. Compared to subjective weights, seven indicators: Risk Factor Control Rate, Intervention Rate, Stroke High-Risk Intervention Rate, Stroke High-Risk Detection Rate, Hypertension Detection Rate, Diabetes Detection Rate, and Dyslipidemia Detection Rate showed increased objective weights. Notably, detection rates for hypertension, diabetes, dyslipidemia, and stroke high-risk populations exhibited the largest average weight increase (227.60%,) followed by intervention rates for previously screened populations and stroke high-risk groups (78.34%), with Stroke Risk Factor Control Rate showing the smallest increase (42.52%). These weight increases indicate high performance variation across Sichuan BHS in stroke risk factor detection, population screening, intervention, and control. Specifically, stroke risk factor detection rates (diabetes: 313.43%, dyslipidemia: 249.25%, and hypertension: 227.61%) demonstrated the largest weight surges, signaling significant variation in detection performance and pronounced disparities in stroke high-risk factor screening implementation among BHS.

The limitations of single weighting methodology are partially illustrated by Follow-up Completion Rate, whose weight dropped from 0.1071 (subjective) to 0.0286 (objective), highlighting potential misalignment between expert assumptions and empirical data [30]. To address such inconsistencies, methodological transparency is essential: subjective methodologies should include consistency verification (e.g., ensuring AHP consistency ratio below 0.1) [31], while objective methodologies must validate data sufficiency [32]. The combined weights: Intervention Rate (0.2303) and Risk Factor Control Rate (0.2207) exemplify effective reconciliation of these inconsistencies, as they integrate theoretical primacy and empirical discriminability, pinpointing indicators that are both conceptually pivotal and practically informative.

### Discrepancies between rankings among BHS under different weighting methodologies

Using the original methodology as a baseline, discrepancies have been observed in BHS ranking across subjective, objective, and combined weighting methodologies, which highlight the critical role of weight determination in multi-criteria decision-making [18]. Under the subjective methodology, BHS A and B maintain their 1st and 2nd ranks, aligning with the original methodology’s recognition of their overall standing. However, other BHS (e.g., D, falling from 4th to 9th; G, rising from 7th to 3rd) exhibit dramatic ranking shifts in their performance evaluations, reflecting the experts’ valued qualitative indicators may be balanced differently by the original methodology. The objective methodology shows even more dramatic changes: BHS D surges from 4th to 1st due to its excellence in indicators with high objective weights (e.g., Intervention Rate, Detection Rates for Hypertension, Diabetes, and Dyslipidemia), while BHS A (falling from 1st to 3rd) and C (falling from 3rd to 4th) drop in rank, as these BHS have inferior performance in such high-objective-weight indicators (e.g., Intervention Rate, Age Deviation Degree, Detection Rates for Hypertension, Diabetes, and Dyslipidemia). This underscores the objective methodology’s sensitivity to statistical patterns over expert priorities [22]. Conversely, rankings from the combined weighting methodology occupy a middle ground, illustrating its role in balancing subjective expertise and objective data. For example, it keeps BHS A and B in the top two (consistent with the original methodology) but adjusts BHS D’s rank from 1st (objective) to 3rd (combined)-a change driven by BHS D’s poor performance in Task Completion Rate and Follow-up Completion Rate, which have the highest and second-highest subjective weights, respectively. This demonstrates that combined weights can reconcile conflicting perspectives by aligning the relative importance of qualitative and quantitative factors. In general, the original methodology implicitly blends qualitative and quantitative considerations to some extent; the subjective and objective methodologies explicitly prioritize either expert-driven or data-driven criteria; and the combined methodology optimizes multi-criteria decision-making by harmonizing these diverse perspectives.

### Practical implications for evaluating BHS performance in the CNSSS program

By integrating subjective and objective weights, we have developed an evaluation model to scientifically assess the performance of BHS in conducting the CNSSS program. Compared to the original methodology (which uses equal weights), the combined weighting methodology has the highest discrimination degree, which means that it has a robust ability to distinguish superior BHS from inferior ones. The combined weighting results highlight the critical importance of three evaluation indicators: Intervention Rate for Previously Screened Populations, Stroke Risk Factor Control Rate, and Intervention Rate for Stroke High-Risk Populations. These findings align with both the objectives of stroke screening and prevention and the core mandates of the CNSSS program. Beyond identifying stroke high-risk individuals in the region, the model emphasizes the need to implement targeted management for these individuals, control preexisting risk factors, and ultimately reduce stroke incidence, recurrence, and mortality.

### Limitations

Several limitations of this study should be acknowledged. Firstly, the objective Entropy Weighting Methodology relies on the premise of indicator independence, which may not hold for inherently correlated indicators such as Hypertension Detection Rate, Diabetes Detection Rate, and Dyslipidemia Detection Rate. Secondly, the sample size of this study was relatively small and geographically limited. The evaluation model was developed based on data from only 16 BHS in Sichuan Province, which may reduce its generalizability to other regions of China. Therefore, caution is advised when extrapolating these findings to broader contexts. Thirdly, the study uses cross-sectional data from 2024, thus longitudinal validation across multiple years is therefore needed to assess the stability of the results.

### Future directions

Future research could address these limitations through the following directions. Firstly, expand the sample size to include BHS from diverse geographical regions. Secondly, incorporate multi-year longitudinal data to enhance temporal validity and verify the long-term stability of the evaluation model. Thirdly, integrate multiple subjective-objective weighting methodologies to further refine the precision of performance evaluation results.

## Conclusions

To our knowledge, this study presents the first novel evaluation model for assessing BHS performance in CNSSS implementation. The findings highlight how weighting methodologies profoundly influence performance rankings, demonstrating that combined weighting effectively balances expert-driven subjectivity and data-driven objectivity. The discrepancy between subjective and objective weights underscores the necessity of transparent, robust multi-methodological evaluation frameworks for public health initiatives. For the CNSSS program, these results provide a scientific basis for refining evaluation systems to prioritize evidence-based interventions and risk factor management. Future research should focus on three key directions: extending this model to nationwide BHS contexts, enhancing its generalizability through sample size expansion and longitudinal data integration, and ensuring robust results through the integration of multiple methodologies.

## Supporting information

S1 File The original de-identified data set. (XLS)
